# A test of frequency‐dependent selection in the evolution of a generalist phenotype

**DOI:** 10.1002/ece3.8831

**Published:** 2022-04-13

**Authors:** Stephanie A. Blain, Louise Chavarie, Mackenzie H. Kinney, Dolph Schluter

**Affiliations:** ^1^ Department of Zoology and Biodiversity Research Center University of British Columbia Vancouver British Columbia Canada; ^2^ 3526 Faculty of Environmental Sciences and Natural Resource Management Norwegian University of Life Sciences Ås Norway

**Keywords:** character displacement, eco‐evolutionary dynamics, frequency‐dependent selection, generalist

## Abstract

A solitary population of consumers frequently evolves to the middle of a resource gradient and an intermediate mean phenotype compared to a sympatric pair of competing species that diverge to either side via character displacement. The forces governing the distribution of phenotypes in these allopatric populations, however, are little investigated. Theory predicts that the intermediate mean phenotype of the generalist should be maintained by negative frequency‐dependent selection, whereby alternate extreme phenotypes are favored because they experience reduced competition for resources when rare. However, the theory makes assumptions that are not always met, and alternative explanations for an intermediate phenotype are possible. We provide a test of this prediction in a mesocosm experiment using threespine stickleback that are ecologically and phenotypically intermediate between the more specialized stickleback species that occur in pairs. We manipulated the frequency distribution of phenotypes in two treatments and then measured effects on a focal intermediate population. We found a slight frequency‐dependent effect on survival in the predicted direction but not on individual growth rates. This result suggests that frequency‐dependent selection might be a relatively weak force across the range of phenotypes within an intermediate population and we suggest several general reasons why this might be so. We propose that allopatric populations might often be maintained at an intermediate phenotype instead by stabilizing or fluctuating directional selection.

## Graphical abstract

Theory for character displacement suggests that a generalist phenotype may evolve via negative frequency‐dependent selection favoring extreme phenotypes in a population. We tested experimentally for negative frequency dependence within generalist populations of threespine stickleback and found only limited support for this form of selection.

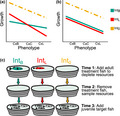

## 1 INTRODUCTION

Populations occurring without close competitors often evolve an intermediate generalist phenotype, in contrast to the divergent specialized phenotypes that evolve via interspecific competition when species are sympatric (Brown & Wilson, 1956; Slatkin, 1980). This pattern, thought to be caused by ecological character displacement, has been observed in numerous traits and taxa (Schluter, 2000; Stuart & Losos, 2013). Examples include intermediate body size in solitary species of *Anolis* lizards in the Lesser Antilles (Losos, 1990), beak depth in the medium beaked ground finch, *Geospiza fortis*, on Daphne Major island in the Galápagos (Grant & Grant, 2014; Schluter et al., 1985), trophic traits in spadefoot toad tadpoles of both *Spea bombifrons* and *S*. *multiplicata* when each occurs alone in southwestern United States ponds (Pfennig et al., 2006), and gill raker length and body shape in solitary lake populations of threespine stickleback (*Gasterosteus aculeatus*) in coastal British Columbia (Schluter & McPhail, 1992).

The form of selection that maintains an intermediate mean phenotype in wild allopatric populations has been little investigated experimentally. In theories of character displacement and of competitive speciation, an intermediate mean phenotype in allopatric populations is maintained via negative frequency‐dependent selection even though an intermediate phenotype is not directly favored by selection (Dieckmann & Doebeli, 1999; Taper & Case, 1992; Wilson & Turelli, 1986). Under this view, those resources consumed by individuals having the most common phenotypes will become depleted most quickly. This will favor individuals having rarer phenotypes that exploit less depleted, alternative resources. If the population is randomly mating and the resource distribution is approximately symmetric, then negative frequency‐dependent selection will result in the maintenance of an intermediate phenotype distribution across generations (Abrams et al., 1993; Kokko & López‐Sepulcre, 2007; Wilson & Turelli, 1986). Therefore, under the hypothesis of negative frequency‐dependent selection, an intermediate phenotype distribution is expected to evolve via an eco‐evolutionary feedback. While several examples of negative frequency‐dependent selection maintaining discrete ecologically relevant phenotypes are known (Benkman, 1996; Bolnick & Stutz, 2017; Hori, 1993; Mappes et al., 2008; Martin, 2016; Pfennig, 1992; Schluter, 2003), less evidence is available that this form of selection can result in the evolution of intermediate distributions of quantitative traits (but see Kusche et al., 2012).

An alternative hypothesis is that intermediate phenotypes in allopatric populations are directly favored regardless of the frequency distribution of phenotypes, perhaps because it allows them to access the broadest possible range of abundant resources. For example, in North American lakes, resource productivity peaks in the littoral zone in spring, and in the pelagic zone in summer (Mittelbach, 1984). An intermediate phenotype would allow a fish population to exploit seasonal resource peaks in turn. Testing for negative frequency‐dependent selection is therefore the first step in distinguishing the two hypotheses. Furthermore, unlike the hypothesis of negative frequency‐dependent selection, the alternative hypothesis requires no feedback. A test of negative frequency‐dependent selection is therefore a test of a theorized eco‐evolutionary feedback in nature, evaluated against an alternative process that involves no feedback.

The present study tested for negative frequency‐dependent selection on a phenotypically variable, intermediate experimental population of threespine stickleback (*Gasterosteus aculeatus*). Sympatric species of threespine stickleback have diverged phenotypically via ecological character displacement along a littoral–pelagic gradient, whereas allopatric populations in otherwise similar lakes are phenotypically and ecologically intermediate (Schluter & McPhail, 1992). Sympatric species pairs are composed of one benthic and one limnetic species which are reproductively isolated from each other, while lakes with allopatric populations have just one stickleback species (Hatfield & Schluter, 1999; Rundle et al., 2000). Within allopatric populations, measures of phenotypes such as body shape and gill rakers are variable and fall between those observed in the benthic and limnetic species, resulting in an intermediate distribution of phenotypes (Svanbäck & Schluter, 2012). Lakes containing sympatric species pairs and those containing allopatric populations of threespine stickleback are similar in their food web characteristics, including resource availability and presence of other fish species, as well as abiotic factors, such as depth and latitude (Ormond et al., 2011; Vamosi, 2003). These populations are all thought to have been founded by marine threespine stickleback between 10,000 and 12,000 years ago as the lakes formed (Taylor & McPhail, 2000). Previous experiments show that negative frequency‐dependent selection between sympatric stickleback species arises via competition for resources (Schluter, 2003). Furthermore, disruptive selection has been observed within some allopatric, phenotypically intermediate populations, which is consistent with frequency dependence but does not directly test for it (Bolnick, 2004; Bolnick & Lau, 2008). Whether selection is frequency dependent within the range of phenotypes present in allopatric, phenotypically intermediate populations is unknown.

**FIGURE 1:**
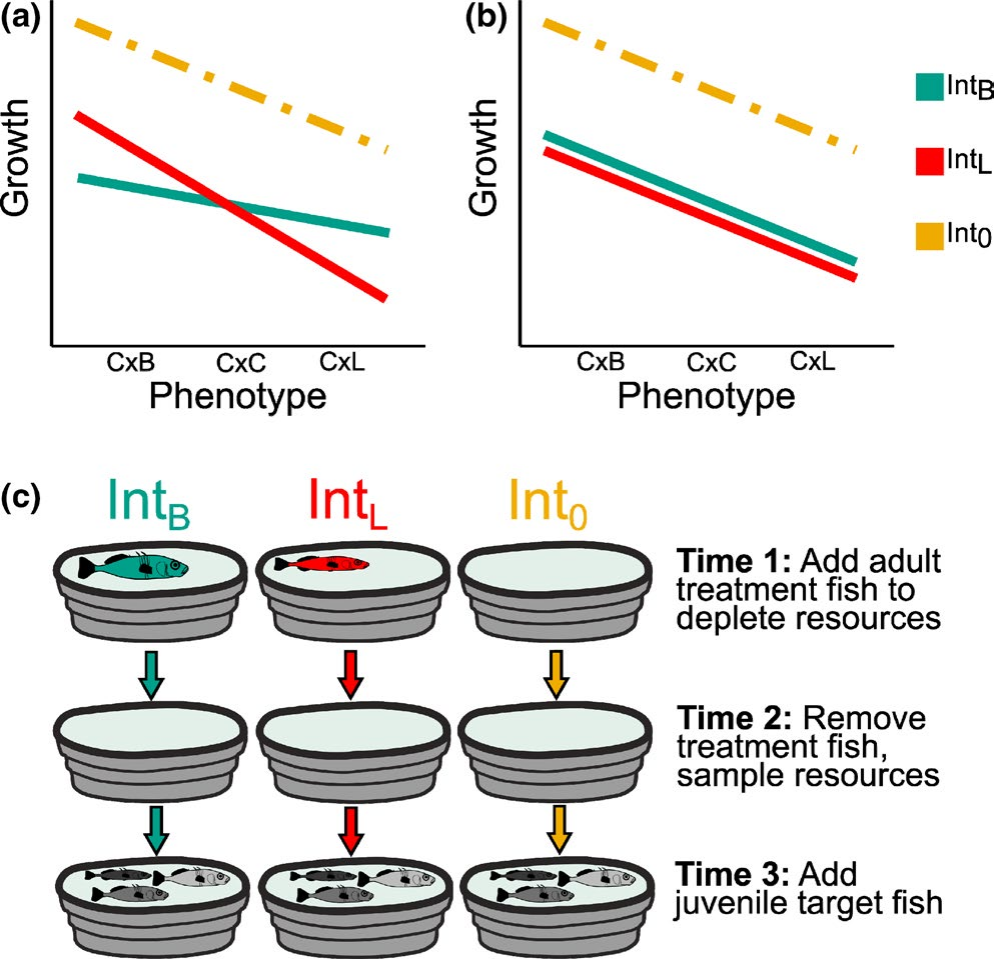
Expectations for growth rate under frequency‐dependent (a) and frequency‐independent selection (b). The lines in the two panels illustrate the expected relationship between phenotype and growth in each mesocosm type – Int_B_ (benthic like treatment), Int_L_ (limnetic like treatment), and Int_0_ (no fish control). Under frequency‐dependent selection (a), the growth of alternate extreme phenotypes is depressed under contrasting Int_L_ and Int_B_ treatments (shown as lines with different slopes). In the absence of frequency‐dependent selection (b), the relationship between phenotype and growth does not depend on treatment phenotype. Mean growth in both treatments is depressed compared with the Int_0_ treatment, in which no fish were added prior to introduction of target fish. (c) Experimental design. There were three main time points in the experiment. At time point 1, four adult treatment fish with benthic‐like (Int_B_) or limnetic‐like (Int_L_) phenotypes were added to each of 40 mesocosms, with 10 left as no fish controls (Int_0_). They were removed at time point 2, and we sampled zooplankton and benthic invertebrates. At time point 3, identical phenotypically variable target populations of 24 juvenile hybrids were added to each mesocosm. We measured the growth and survival of these experimental target fish

We tested the prediction of negative frequency‐dependent selection according to an eco‐evolutionary feedback within intermediate phenotype distributions. To do so, we manipulated the phenotype distribution of stickleback populations in mesocosms, creating one treatment population that was more limnetic like and one that was more benthic like (Figure 1). We then measured the effect of the two phenotype distribution treatments on the growth and survival of a phenotypically variable intermediate target population. Zooplankton and benthos, which are common threespine stickleback prey, were additionally measured to test the expectation that the two phenotype distribution treatments would differentially deplete resources. This would cause changes in invertebrate community composition that would be expected to have phenotype‐dependent impacts on target population growth and survival (Best et al., 2017; Matthews et al., 2016). If selection was frequency dependent, then altering the frequency of phenotypes was predicted to affect individuals with similar phenotypes most negatively in the experimental target population (Figure 1). If selection was not frequency dependent, then the performance of different phenotypes in the experimental target population would be affected by the presence of treatment fish, but not their distribution of phenotypes.

## 2 METHODS

### 2.1 Experimental design

**FIGURE 2:**
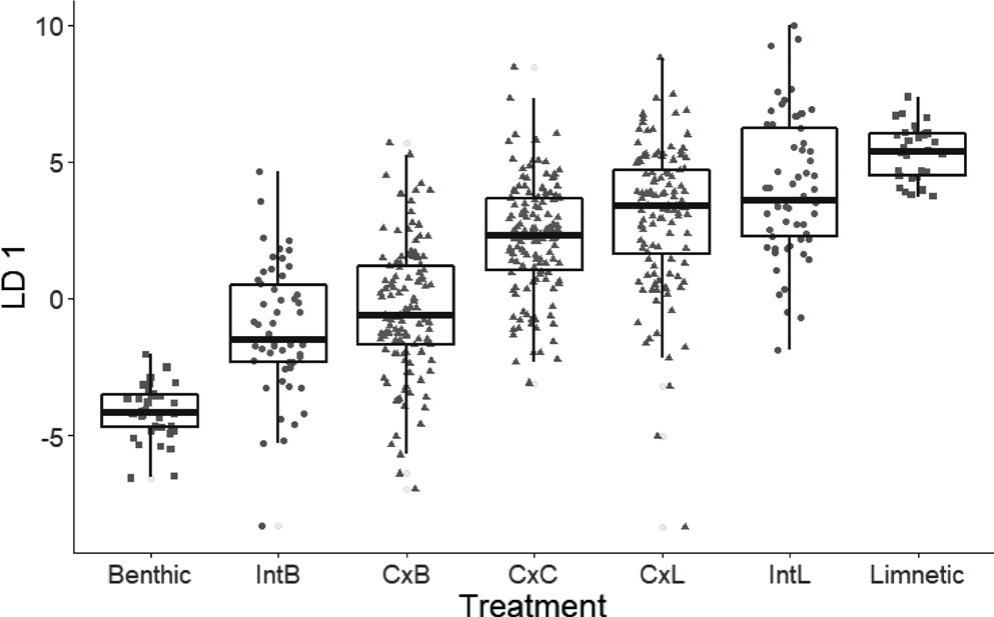
Position of different experimental fish phenotypes along a linear discriminant axis. Each point represents one individual. Benthic and limnetic individuals are from the species pair populations in Priest and Paxton Lakes (squares), the Int_B_ and Int_L_ individuals were the fish used in the treatment phase of the experiment (circles), and the C × B, C × C, and C × L individuals were the experimental target population (triangles). All target population individuals are from the individually marked dataset. Body shapes were quantified after the experiment, so individuals included in this figure were only those that survived the experiment

The experiment was performed in mesocosms with two distinct stages, a treatment stage and a response stage, following Matthews et al. (2016; Figure 1). Although the phenotype distributions of natural allopatric stickleback populations are generally unimodal and intermediate to the extreme benthic and limnetic phenotypes of the species pairs, their mean phenotypes are variable among lakes. Due to differences in lake size and community composition, some populations exhibit more benthic‐like characteristics, such as few gill rakers, and others showing more limnetic‐like characteristics, such as a streamlined body shape (Bolnick & Ballare, 2020; Miller et al., 2015). We exploited this variation to generate contrasting experimental treatments with more benthic‐like (“Int_B_ treatment”) or more limnetic‐like (“Int_L_ treatment”) phenotype distributions (Figure 2). We chose to generate Int_B_ and Int_L_ treatments using allopatric populations with more benthic‐ or limnetic‐like means rather than using the more phenotypically distinct benthic and limnetic species in order to include phenotypes within the range expected in an intermediate generalist population. In the treatment stage, which began in September 2017 and lasted 1 month, four adult stickleback from either an Int_B_ or Int_L_ treatment were added to a total of 40 mesocosms. Ten mesocosms had no fish added during the treatment stage (“Int_0_” treatment). The phenotype frequency distributions were therefore manipulated in the treatment phase (the first month of the experiment). After a month, we removed the treatment fish and sampled zooplankton and benthic invertebrates to test for the impact of treatment on resource communities in the two main habitats. If frequency‐dependent selection occurred, mediated by an eco‐evolutionary feedback, then the resource communities present after the treatment phase was predicted to depend on the phenotypes of treatment population fish.

In the second stage of the experiment, replicate phenotypically variable experimental target populations of 24 juvenile fish were tagged using elastomers then added to each mesocosm in October 2017. Growth rate and survival were measured in these juveniles as proxies for fitness, after their removal in December 2017. Growth rate is linked to feeding performance and fecundity in sticklebacks (Arnegard et al., 2014; Bolnick & Lau, 2008; Schluter, 1995). The experimental setup therefore mimics a scenario in which adults of one generation impact juveniles of the next generation. The prediction under frequency dependence was that performance of a given target population phenotype would depend on the phenotype distribution present in the treatment phase.

### 2.2 Study populations

Treatment and target population fish came from four types of lake stickleback populations: (1) allopatric with an intermediate phenotype distribution, (2) allopatric with a more limnetic‐like phenotype distribution, (3) allopatric with a more benthic‐like phenotype distribution, and (4) sympatric benthic and limnetic species pairs. We use the term “species” to refer to sympatric pairs of reproductively isolated and ecologically distinct benthic and limnetic species, and the term “populations” to refer to separate populations that would potentially interbreed if they came into contact with each other. Accurately assessing the position of phenotypically intermediate stickleback along a benthic‐to‐limnetic phenotypic axis is challenging to do accurately while individuals are still alive. We therefore relied on known differences in mean phenotypes of stickleback from different allopatric populations to generate Int_B_ and Int_L_ treatments. There is a relatively high level of variability within these allopatric populations, which lead to variation that we could not control in the degree to which treatment phenotypes were more or less limnetic‐like or benthic‐like (Figure 2). Adult treatment fish were collected from the wild between April and June 2017 and held in aquaria in the University of British Columbia aquatics facility until their introduction into the mesocosms in September 2017. Stickleback for the Int_L_ treatment populations were caught by minnow trap and dip net in Ambrose, North, and Garden Bay lakes.

Due to a shortage of individuals resulting from mortality in the lab prior to the start of the experiment, Int_L_ treatment populations were supplemented with individuals from the limnetic species from Little Quarry and Priest lakes, in 6 of the 20 limnetic‐like treatment mesocosms. These individuals were also wild caught in April and May 2017 and held in aquaria until September 2017. Resource depletion did not differ among Int_L_ mesocosms with different source populations. The sampled invertebrate biomass (see Section 2.4) was similar between mesocosms that contained limnetics and those that contained limnetic‐like intermediates for both zooplankton (*F*_1,18_ < 0.01, *p* = .98) and benthos (*F*_1,18_ = 1.23, *p* = .28). Fish for the Int_B_ populations were caught by minnow trap in Hoggan and Bullock lakes. Four Int_L_ individuals were added to each of 20 mesocosms and four Int_B_ individuals were added to another 20 mesocosms. This number of individuals was chosen because populations of four individuals were sufficient to differentially deplete resources in mesocosms in past experiments (Harmon et al., 2009; Rudman & Schluter, 2016).

After the experiment, we used body shape, which varies in a repeatable way between benthic and limnetic stickleback and correlates to resource acquisition (Gow et al., 2008; Schluter, 1995), to verify that Int_L_ and Int_B_ treatment population stickleback used and retrieved from the experiment were indeed either more benthic like or more limnetic like. Each recovered fish was stained with alizarin red and photographed. An additional set of wild caught stickleback of the sympatric benthic and limnetic species from Priest and Paxton Lakes were stained and photographed for comparison. A total of 22 landmarks were used on each fish using the program tpsDig2 v 2.31 (Rohlf, 2018), following the landmarks used in Ingram et al. (2012). A Procrustes analysis on the x and y coordinates of each landmark was performed using the “geomorph” package in R v 4.0.3 (Adams & Otárola‐Castillo, 2013; R Core Team, 2020). A linear discriminant analysis was performed on the scaled and aligned coordinates corresponding to the benthic and limnetic fish using the “MASS” package (Venables et al., 2019). Linear discriminant axis one therefore represented a benthic‐to‐limnetic phenotypic axis. Treatment fish were then projected onto this axis (Figure 2).

We exploited among‐population variation along a benthic–limnetic phenotypic axis to construct an experimental target population with high phenotypic variance. The target fish population was a mixture of eight individuals from each of three cross types: (1) Cranby Lake females crossed to Paxton Lake limnetic males (C × L juveniles), (2) Cranby Lake females crossed to Paxton Lake benthic males (C × B juveniles), and (3) Cranby Lake females crossed to Cranby Lake males (C × C juveniles) (see Section 2.5 below for more details on the crossed juveniles). Cranby Lake is located near Paxton Lake and contains an allopatric population that is phenotypically intermediate between the benthic and limnetic species. This crossing scheme allowed us to generate an intermediate population with a wide phenotype distribution (Figure 2). We chose to use a target population with inflated phenotypic variation to increase the sensitivity with which we could measure selection (Schluter, 1994). A larger sample size was used for the target population than for the treatment population to account for the smaller biomass of juveniles and to allow for competition among individuals even with some mortality.

### 2.3 Mesocosm construction and treatment

Experimental mesocosms were constructed outdoors in 50 cattle tanks. The mesocosms had a volume of 1136 L, a depth of 64 cm, and a width of 175 cm. In May 2017, we added 12.5 kg dry weight of sand to the bottom of each mesocosm and filled them with water. Each mesocosm was seeded with zooplankton from adjacent experimental ponds and with mud containing benthic invertebrates from a nearby reservoir pond. The mesocosms were left unmanipulated from June to August 2017, giving insects with an aquatic larval stage an opportunity to lay eggs in the tanks. To provide nutrients to stimulate phytoplankton growth, we added 0.976 g KNO_3_ and 0.067 g KH_2_PO_4_ to each mesocosm in August 2017.

During the experiment, mesocosms were surveyed daily for mortalities, which were removed and replaced with a fish from the same population type (Int_B_ or Int_L_) to maintain a density of four fish per mesocosm. After the month‐long treatment phase, treatment fish were removed by minnow trap and dip net over a 2‐week period. All treatment population individuals were recovered in 24 of the 40 treatment mesocosms, and between zero and three individuals were recovered in the remaining 15 mesocosms. The decision was made nonetheless to proceed with adding the target fish as we assumed that these individuals had died in the substrate at the bottom of the tank or were eaten by predatory birds or insects and were not recoverable without creating undue disruption to the mesocosms. The timing of these assumed deaths during the experiments is unknown. Results with all mesocosms included are presented in the main text, and results from only tanks where all four fish were recovered are included in Supplementary materials. The direction of results is consistent between both datasets, with some differences in statistical significance given differences in sample size (see Section 3, Tables S1 and S2).

### 2.4 Benthic invertebrate and zooplankton sampling and analysis

Between the first and second stages of the experiment, four zooplankton samples were taken through the water column in each cattle tank using a 5.08‐cm‐diameter PVC pipe with a tennis ball attached to a rope that could be pulled in to act as a stopper. Samples were stained and preserved in iodine. They were later identified to a taxonomic level ranging from family to subclass and the length was measured using an ocular micrometer in a dissecting microscope. We used data on Daphniidae as well as Calanoid and Cyclopoid copepods to represent pelagic resource availability (Schluter & McPhail, 1992). Length measurements of Daphniidae and Copepoda specimens were used to estimate biomass, using length–weight regressions from Dumont et al. (1975). Biomass estimates were not normally distributed, so they were ln‐transformed.

Two 120 cm^2^ samples of benthic substrate were taken using a dip net from standardized locations in each mesocosm – one near the mesocosm edge and one near the center. The full depth of substrate was sampled at each location. Samples were searched by hand for benthic invertebrates for up to 20 mins, immediately after collections. Benthic invertebrates were preserved in ethanol, and later identified and measured using an ocular micrometer in a dissecting microscope. Identification ranged from a family to a class level and length measurements were converted to biomass using published length–weight regressions (Baumgärtner & Rothhaupt, 2003; Benke et al., 1999; McKinney et al., 2004; Miyasaka et al., 2008).

The benthos and zooplankton biomass estimates were each divided by the surface area of the sample taken, so that all estimates were in μg/cm^2^. We calculated the total biomass (μg/cm^2^) as the sum from each mesocosm. We then log‐transformed each biomass estimate after adding the constant to 0.1 to allow zero values to be included in the dataset. The data were not normally distributed (Shapiro–Wilk normality test: *W* = 0.96, *p* = .002), so we used a two‐group Mann–Whitney U test to determine whether invertebrate biomass in each mesocosm depended on fish presence/absence treatment (Int_0_ vs. Int_L_/Int_B_).

We predicted that Int_B_ and Int_L_ fish would more efficiently deplete benthos and zooplankton, respectively. To test this, we first converted sample type to a numeric value (benthos = 0, zooplankton = 1) and calculated the slope of log‐transformed biomass against sample type for each mesocosm. We then used a two‐group Mann–Whitney U test on the slopes between treatments under the alternative hypothesis that the slope between sample type and biomass was greater in Int_B_ than Int_L_ mesocosms.

To test for shifts in community composition in invertebrate communities, we first divided counts of individuals per taxonomic category by the surface area of the sample taken, then calculated Bray–Curtis distances between tanks using the “vegan” package in R (Oksanen et al., 2020). We then evaluated the effect of treatment fish presence/absence (Int_0_ vs. Int_L_/Int_B_) and treatment fish phenotype (Int_L_ vs. Int_B_) on those distances using the function “adonis()” which conducts a multivariate analysis of variance using distance matrices (Anderson, 2001; Oksanen et al., 2020). To visualize these distances, we used non‐metric multidimensional scaling (NMDS) with four dimensions. We then used linear models to test whether there was a difference among treatments along any of those four axes.

### 2.5 Target juvenile stickleback population

C × L, C × C, and C × B crosses were performed throughout May 2017 in the field and then transported to the UBC aquatics facility to be hatched and raised in aquaria. Crosses were performed by mixing eggs from one gravid Cranby Lake female with one crushed testis from a Paxton limnetic, Paxton benthic, or Cranby male. They were held in aquaria until transportation to the mesocosms. For 10 Int_L_, 10 Int_B_, and 5 Int_0_ mesocosms, fish were individually marked with elastomer tags to identify their cross type and allow measurement of individual growth rates. Due to logistical constraints, in the other 25 mesocosms, C × C juveniles were batch marked with elastomer tags by giving the same type of elastomer tag to each fish. Mesocosms were assigned randomly to contain individually or batch‐marked populations. C × L juveniles and C × B juveniles were the most morphologically distinct cross types, so these fish were left unmarked. The individually marked and batch marked fish required different methods of analysis. For mesocosms with individually marked fish, the fish is the sampling unit (nested within mesocosm). Including batch marked fish required using the mesocosm as the sampling unit, with an average growth change calculated for each cross type in each mesocosm.

At the end of the experiment, C × L juveniles and C × B juveniles retrieved were identified by a discriminant function analysis of their overall body shape, using the same landmarks used for treatment population fish. We performed a linear discriminant analysis on the scaled and aligned coordinates for individually marked fish of known cross type. The results of this analysis were used to classify remaining individuals. Individuals not assigned to a cross type with posterior probability higher than 95% were removed from later analyses.

### 2.6 Growth and survival estimates

Standard lengths were measured from photographs of target population fish taken before introduction to and after removal from mesocosms, using the program ImageJ (Schneider et al., 2012). The photographs were taken of the left side of each fish with a ruler in the frame of the photo. Each fish was also weighed at both time points by placing the fish in a tupperware container with water on a zeroed scale. Growth was calculated for all individually marked fish as the natural log of measured length and weight at the end of the experiment minus the natural log of the same measurements at the beginning of the experiment. For all mesocosms, we calculated average change in length for each cross type. This was calculated as the mean length of fish of a cross type in a mesocosm at the end of the experiment minus the mean length of fish of a cross type in a mesocosm at the beginning. Whether or not individuals survived could be determined for the individually marked fish only. There were, therefore, four different response variables: (1) length change in individually marked fish, (2) weight change in individually marked fish, (3) survival of individually marked fish, and (4) length change in batch marked and individually marked fish.

### 2.7 Treatment fish presence/absence effects

To evaluate the predicted effect of treatment fish presence/absence in each of the three response variables, we tested for a difference in each mean growth and proportion survived between Int_0_ mesocosms, where treatment fish were absent, and mesocosms where treatment fish were present (Int_L_ and Int_B_). We used a Welch's two‐sample *t*‐test with the alternative hypothesis that growth in Int_0_ mesocosms was greater than in Int_L_ and Int_B_ mesocosms. We estimated standardized effect sizes with Cohen's *D*. Cohen's *D* values near 0.2 and 0.5 are generally considered to be small and moderate, respectively, while an effect size of 1.2 is considered very large (Sawilowsky, 2009).

We additionally tested whether the presence of treatment fish affected the slope of the relationship between target fish phenotype and outcome (specifically weight change, length change, and proportion survival). To do this, we followed the methods outlined below for comparisons between slopes in Int_L_ and Int_B_ mesocosms (see “Tests of Selection”) but instead compared mesocosms where treatment fish were absent (Int_0_) and present (Int_L_ and Int_B_). Because this did not address any of our predictions for the experiment, these results are included in the Supplement (Table S3). Slopes of regressions of survival on body shape along the benthic–limnetic axis tended to be larger in treatment fish absence mesocosms than in treatment fish presence mesocosms (Table S3). In several comparisons, the slopes of regressions of growth (weight and length) on benthic–limnetic body shape were smaller in treatment fish absence mesocosms than in treatment fish presence mesocosms (Table S3).

### 2.8 Tests of selection

For mesocosms with individually marked fish, we estimated the slope of the relationship between LD1 (which corresponded to an axis of body shape from benthic like to limnetic like) and each length and weight change. These slopes were expected to be non‐zero due to intrinsic differences in growth rates among stickleback phenotypes (Figure 1; Hatfield & Schluter, 1999). We then tested whether slopes from Int_L_ mesocosms are less than those from Int_B_ mesocosms using a Welch's two‐sample *t*‐test. If selection was negative frequency dependent, we would expect fish with more limnetic‐like phenotypes (i.e., C × L fish) to exhibit higher growth in Int_B_ relative to Int_L_ mesocosms (Figure 1). This would correspond to a more negative slope between growth and body shape in Int_L_ than Int_B_ mesocosms. We then repeated this test with cross type converted into numeric values (C × B = −1, C × C = 0, C × L = 1) as the predictor instead of LD1.

For fish from all mesocosms (individually marked and batch marked), we calculated the mean length and mean LD1 for the three cross types from each mesocosm then calculated a slope between those variables for each mesocosm. We used a Welch's two‐sample t‐test to evaluate whether slopes from Int_L_ mesocosms were less than slopes from Int_B_ mesocosms. This test was repeated with cross type converted into numeric values as the predictor for each slope.

For survival, we first calculated the mean LD1 and proportion survived for the three cross types from each Int_L_ and Int_B_ mesocosm. We calculated a slope for each mesocosm using these three points, then evaluated whether the slopes in Int_L_ mesocosms were less than those in Int_B_ mesocosms using a one‐sided Welch's two‐sample *t*‐test. We then repeated this test with cross type converted to numeric values as the predictor.

## 3 RESULTS

### 3.1 Invertebrate biomass response

**FIGURE 3:**
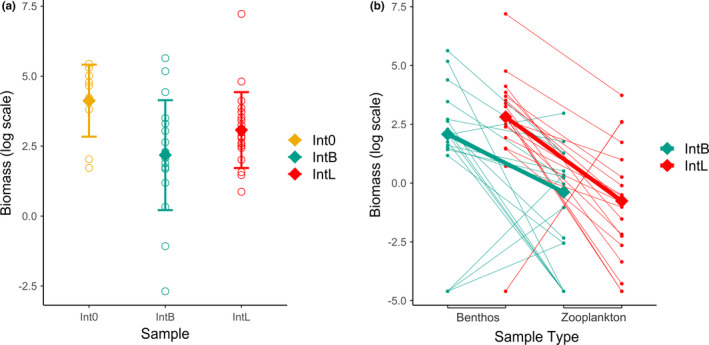
(a) Total invertebrate biomass. Circles represent the total biomass (μg/cm^2^) of invertebrates sampled from a mesocosm. Diamonds represent medians, while error bars represent 1 standard deviation. On the *Y*‐axis, biomass is given on a natural log scale. (b) Invertebrate biomass by habitat. Points represent the total biomass (μg/cm^2^) of invertebrates sampled from a mesocosm on a log scale, with lines joining biomass estimates from the same mesocosm. Diamonds represent medians for each sample type from each Int_B_ and Int_L_ mesocosms

Invertebrate community biomass, sampled after treatment fish were removed and before the addition of the experimental target population, was greater overall in control (Int_0_) than fish addition (Int_B_ and Int_L_) mesocosms (Figure 3a; *W* = 301, *p* < .01), confirming food resource depletion in the presence of fish. The slope of regressions of biomass on invertebrate sample type differed slightly in the predicted direction between Int_B_ and Int_L_ mesocosms and not significantly (Figure 3b; *W* = 218, *p* = .44).

Invertebrate community composition differed between the control (Int_0_) and fish addition treatment (Int_B_ and Int_L_) mesocosms (multivariate ANOVA: *F*_1,47_ = 2.64, *p* < .01), indicating an effect of resource depletion in the presence of fish. In contrast to the first prediction from the frequency dependence hypothesis, we did not detect a difference in community composition between Int_B_ and Int_L_ mesocosms (multivariate ANOVA: *F*_1,37_ = 0.95, *p* = .47). Int_0_ was differentiated from Int_L_ and Int_B_ along the third NMDS axis (Figure S1; *F*_2,46_ = 16.76, *p* < .01), but treatment groups did not vary along the first (*F*_2,46_ = 0.08, *p* = .93), second (*F*_2,46_ = 0.18, *p* = .83), or fourth axes (*F*_2,36_ = 2.39, *p* = .10).

### 3.2 Survival among experimental target fish

**FIGURE 4:**
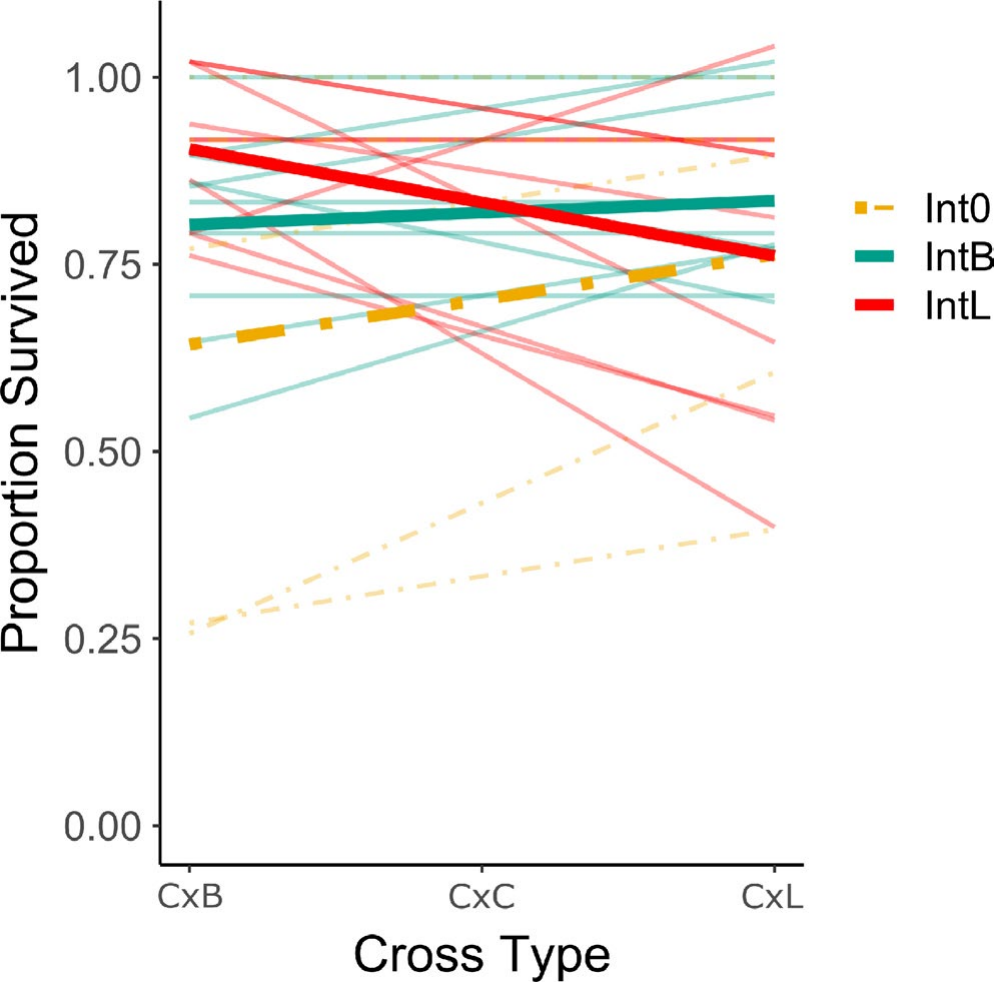
Relationship between survival and cross type in contrasting treatments. Cross was converted to a numeric value, with C × B = −1, C × C = 0, and C × L = 1. Each thin line represents the relationship between growth and cross type in one mesocosm while bold lines represent the mean slopes for mesocosms with each treatment

Mean survival of experimental target fish was similar between mesocosms in which treatment fish had been present and absent (Figure 4; *t*_4.53_ = −0.9, *p* = .42, Cohen's *D* = −0.54). As predicted under negative frequency‐dependent selection, the slope of regressions of survival on cross type differed between treatments (Figure 4; *t*_14.69_ = 2.34, *p* = .03, Cohen's *D* = 1.05). The limnetic‐like treatment (Int_L_) reduced survival of the most limnetic‐like experimental fish relative to the most benthic‐like experimental fish. Conversely, the benthic‐like treatment (Int_B_) reduced survival of the most benthic‐like target fish relative to the most limnetic‐like target fish. The same direction of difference was observed for slopes of regressions relating survival to body shape of experimental fish (Figure S2; *t*_16.89_ = 1.96, *p* = .07, Cohen's *D* = 0.89).

### 3.3 Growth rates among experimental fish

**FIGURE 5:**
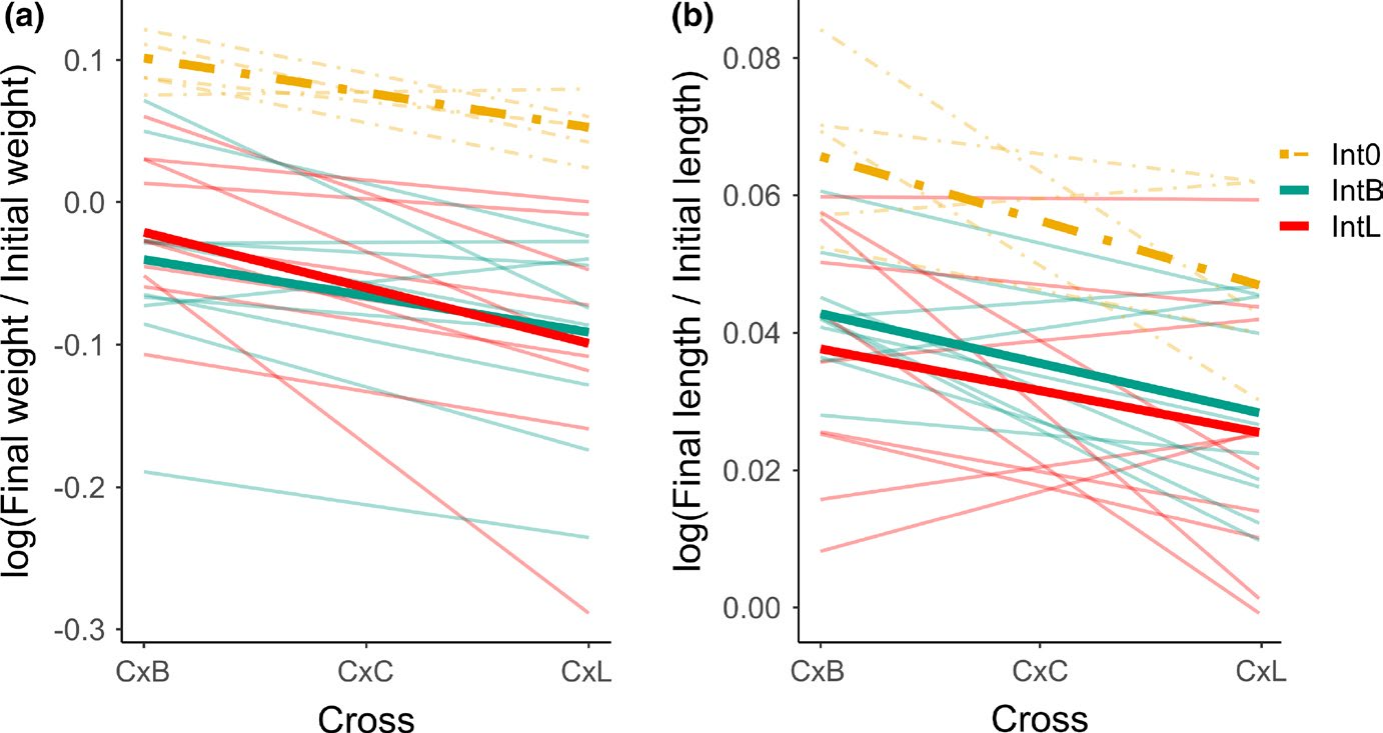
Relationship between growth, measured by weight (a) and length (b), and cross type in contrasting treatments. Cross was converted to a numeric value, with C × B = −1, C × C = 0, and C × L = 1. Each thin line represents the relationship between growth and cross type in one mesocosm while bold lines represent the mean slopes for mesocosms with each treatment

Food depletion by treatment population fish impacted experimental target fish growth. Mean growth of individually marked fish was highest in Int_0_ mesocosms (treatment fish absent) when measured by weight change (Figure 5a; *t*_823_ = 8.89, *p* < .01, Cohen's *D* = 3.01) and length change (Figure 5b; *t*_9.05_ = 4.99, *p* < .01, Cohen's *D* = 2.19). The result was the same for length change in batch‐marked fish (Figure S3; *t*_16.62_ = 2.8, *p* = .01).

Slopes of regressions of growth rate on cross type differed weakly in the predicted direction between frequency treatments (Int_B_ and Int_L_) for weight change in individually marked fish (Figure 5a; *t*_16.93_ = 1.25, *p* = .23, Cohen's *D* = 0.56) and length change in batch‐marked fish (Figure S3A; *t*_35.87_ = 0.78, *p* = .44, Cohen's *D* = 0.25). For length change in individually marked fish, differences in slopes of the relationship between growth and cross type were very small and not in the predicted direction (Figure 5b; *t*_14.32_ = −0.04, *p* = .97, Cohen's *D* = −0.02).

Slopes of regressions of growth rate on body shape differed slightly between frequency treatments (Int_B_ and Int_L_) but not in the predicted direction when measured in individually marked fish by weight change (Figure S4A; *t*_16.95_ = −0.84, *p* = .41, Cohen's *D* = −0.38) and length change (Figure S4B; *t*_16.91_ = −0.33, *p* = .74, Cohen's *D* = −0.15). Slopes of regressions of growth rate on body shape were weakly different in the predicted direction when measured by length change in batch‐marked fish (Figure S3B; *t*_32.15_ = 1.09, *p* = .28, Cohen's *D* = 0.36).

## 4 DISCUSSION

When a randomly mating population evolves on a symmetric resource gradient, resource competition is predicted to result in frequency‐dependent selection leading to the evolution of an intermediate phenotype (Dieckmann & Doebeli, 1999; Taper & Case, 1992). Alternatively, selection might directly favor an intermediate phenotype without frequency‐dependent selection. We carried out an experimental test of frequency‐dependent selection via an eco‐evolutionary feedback using intermediate populations of threespine stickleback and detected only weak effects. Survival selection was weakly frequency dependent. The direction of estimates was variable when growth was used as a fitness metric and point estimates were small and uncertain. Resource depletion occurred with detectable effects on growth, suggesting that competition for food was nevertheless present. We conclude that frequency‐dependent selection is likely to be present, but if so, it is not strong.

Aspects of the experimental conditions warrant caution in drawing conclusions about the role of frequency‐dependent selection on stickleback populations. Performing the experiment in mesocosms might have restricted the width of the resource gradient, such as by having a limited pelagic zone. Character displacement theory shows that a narrow resource gradient weakens frequency‐dependent selection (Dieckmann & Doebeli, 1999; Taper & Case, 1985). Furthermore, this experiment was run on a short time frame. It is possible that a longer period of resource depletion would be required to generate a noticeable impact of the different phenotypes on the environment. This also means that only one part of the target population's life cycle was measured, so stronger effects may have emerged if there was more time for juvenile growth or if effects were measured over multiple generations. Additionally, adult sticklebacks were used as a treatment population, whereas juvenile sticklebacks were used as a target population. Given that adult and juvenile stickleback have differences in morphology and gape width, it is possible that they would consume resources differently. As a result, it is possible that frequency dependence would only be observed among individuals of the same age class. Despite the caveats, we have shown that frequency‐dependent selection, if present within this range of phenotypes, is not always strong and easily detectable. Although this is not the final word on frequency dependence in this system, we nevertheless suggest that the results have interesting implications for our understanding of the evolutionary processes acting in intermediate populations.

Our results are somewhat surprising because they seem at odds with theory for trait evolution along a resource gradient in the presence of competition (Roughgarden, 1976; Taper & Case, 1985). They are additionally puzzling because frequency‐dependent selection has been detected between sympatric species of threespine stickleback differing in mean phenotype (Rundle et al., 2003; Schluter, 1994, 2003). However, under existing theory, frequency‐dependent directional selection is expected to weaken with greater similarity of competing individuals (Schluter, 2000). Therefore, differences between sympatric and allopatric populations might prevent similar intensities of selection from occurring in both contexts. At the start of the character displacement process in stickleback, the phenotype distribution in lakes containing two sympatric species is thought to have been broader overall than that in single‐species, allopatric populations (Svanbäck & Schluter, 2012; Taylor & McPhail, 2000). Phenotypes within intermediate populations might always overlap significantly in resource use, or the overlap between limnetic‐like and benthic‐like phenotypes might be higher when each occurs in the absence of alternative phenotypes. Variation in resource use within and among intermediate populations may therefore not be large enough to exert detectably different ecological impacts or to generate an eco‐evolutionary feedback, and therefore frequency‐dependent selection. A broader phenotype distribution than that found within populations may be necessary to generate strong frequency dependence in stickleback.

Another possible explanation for our finding of weak selection is that the resource distribution was too narrow in mesocosms relative to the breadth of resources utilized by consumers. For strong frequency dependence driven by an eco‐evolutionary feedback to emerge, resource distributions must be wide enough for individuals with uncommon phenotypes to have undepleted resources to access (Dieckmann & Doebeli, 1999; Rainey & Travisano, 1998). For a population of individuals exploiting most of the resources in an environment with a narrow resource distribution, this may not be the case. Stickleback populations are most phenotypically variable and most commonly experience disruptive selection (another possible outcome of frequency‐dependent selection – see below) in intermediate‐sized lakes with relatively equal ratios of benthic‐to‐limnetic habitat (Bolnick & Ballare, 2020; Bolnick & Lau, 2008). These may therefore be the habitats in which frequency dependence within intermediate populations is strongest and most likely to be detected. Nonetheless, previous experiments have shown that phenotypically divergent stickleback cause divergent ecosystem effects in mesocosms, and that these ecosystem effects can generate eco‐evolutionary feedbacks (Des Roches et al., 2013; Harmon et al., 2009; Matthews et al., 2016; Rudman & Schluter, 2016). Those experiments, however, used a wider distribution of phenotypes with greater differences between phenotype treatments. Weak or absent frequency‐dependent selection could instead be a consequence of the way in which phenotypes deplete resources, and the degree of overlap between them. If individuals within intermediate stickleback populations consume a broader or more plastic range of resources, then individuals with different phenotypes may exhibit more overlap in resource use. This would mean that increasing the frequency of one phenotype would impact other phenotypes more or less equally, leading to a lack of strong frequency dependence.

A prediction of the same theory, which we did not test here, is that selection on intermediate populations should be disruptive (Wilson & Turelli, 1986). Surveys and field experiments have found that selection is variable and sometimes disruptive in single‐species populations of threespine stickleback, depending on lake characteristics, and that the strength of disruptive selection is density dependent (Bolnick, 2004; Bolnick & Lau, 2008). However, in those lakes where disruptive selection does occur it also tends to be quite weak (Bolnick & Lau, 2008). Disruptive selection has been detected in an experimental pond population of F_2_ hybrids between sympatric benthic and limnetic species (Arnegard et al., 2014). In both cases, disruptive selection could have been generated by either frequency dependence or a bimodal resource distribution (Rueffler et al., 2006; Wilson & Turelli, 1986). In phenotypically intermediate populations of *S*. *multiplicata* spadefoot toads, which are another set of allopatric populations from a character displacement series, disruptive selection is present and generated by competition between phenotypically similar individuals, as predicted by character displacement theory (Martin & Pfennig, 2009). The present experiment demonstrated that frequency dependence is hard to detect even with the inflated variance of our target experimental populations. We thus suggest that frequency‐dependent selection may be present, but weak within the limited range of phenotypes in allopatric populations.

Our findings are broadly consistent with a particularly well‐studied intermediate natural population, the medium ground finch *G*. *fortis* on Daphne Major Island in the Galàpagos. Mean beak size in this population is intermediate between the means of the small and medium ground finch species that occur in sympatry on most other islands (Schluter et al., 1985). Decades of field study have shown that on Daphne Major, selection on *G*. *fortis* is typically directional and varies in direction and strength from year to year. The net effect is to maintain the population at an intermediate phenotype (Grant & Grant, 2014; Schluter et al., 1985). The fluctuating selection and resulting evolution are closely tied to annual variation in environmental factors, particularly rainfall (Grant & Grant, 2014; Nosil et al., 2018). This suggests that frequency‐dependent selection within the range of phenotypes in the population might not be the main cause of an intermediate phenotype in the allopatric *G*. *fortis* population, although this has not been tested experimentally. Given the results of the present experiment along with weak and spatially varying disruptive selection in allopatric populations (Bolnick, 2004; Bolnick & Lau, 2008), the same might be true in stickleback.

## CONFLICT OF INTEREST

None.

## AUTHOR CONTRIBUTIONS

**Stephanie A. Blain:** Conceptualization (equal); Data curation (lead); Formal analysis (lead); Investigation (lead); Methodology (equal); Visualization (lead); Writing – original draft (lead); Writing – review & editing (equal). **Louise Chavarie:** Data curation (supporting); Formal analysis (supporting); Investigation (supporting); Supervision (supporting); Visualization (supporting); Writing – review & editing (equal). **Mackenzie H. Kinney:** Investigation (supporting); Methodology (supporting); Writing – review & editing (supporting). **Dolph Schluter:** Conceptualization (equal); Formal analysis (equal); Funding acquisition (lead); Methodology (equal); Project administration (equal); Resources (lead); Supervision (lead); Writing – review & editing (lead).

## Supporting information

Supplementary Material

## Data Availability

Sampled invertebrate lengths and IDs, experimental fish pre‐ and post‐experiment measurements, and shape data for all fish (treatment and experimental) are available on dryad at: https://doi.org/10.5061/dryad.qv9s4mwgr.
