# Supplementary material for: A test of frequency‐dependent selection in the evolution of a generalist phenotype
Source: Ecol Evol. 2022 Apr 13;12(4):e8831. doi: 10.1002/ece3.8831 (PMC9006234; doi:10.1002/ece3.8831)
Supplement: Supplementary file 1 — Supplementary Material [file ECE3-12-e8831-s001.docx]

**Supplementary Figures**


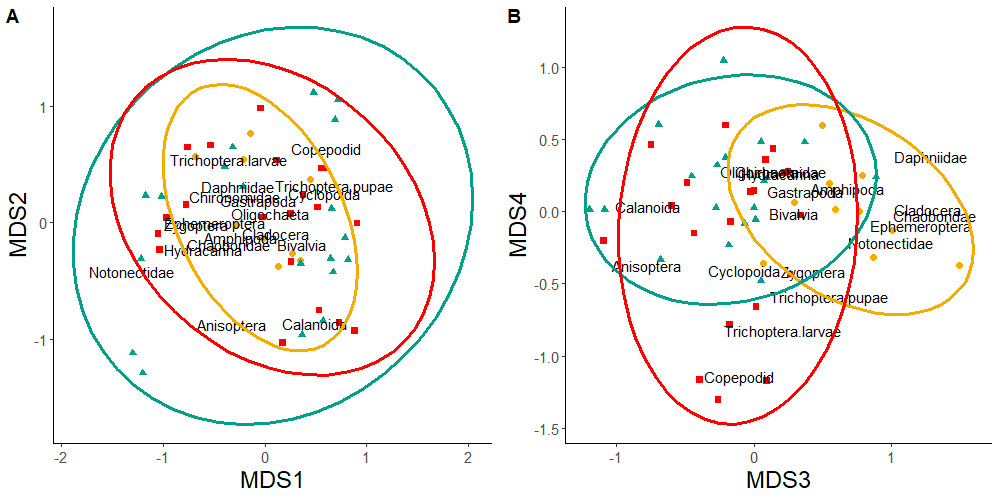


*Fig. S1.* NMDS of invertebrate community composition. Each point represents one mesocosm, with colour indicating treatment (Int_0_ = yellow circle, Int_B_ = green triangle, Int_L_ = red square). Ellipses represent a 95% confidence interval for each treatment. Labels indicate the ordination of invertebrate taxa.


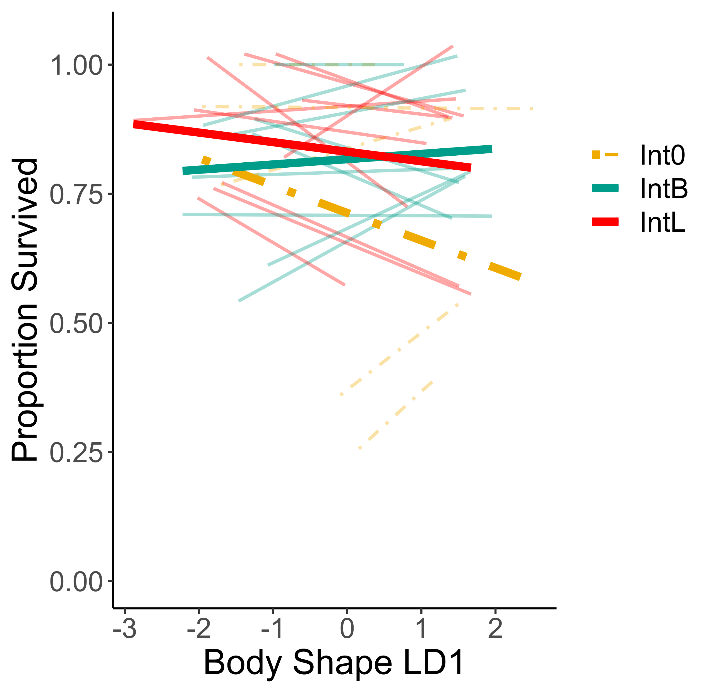


*Fig. S2.* Target population survival in contrasting frequency distributions of phenotypes of individually-marked fish. Each line represents one mesocosm. More negative LD1 values indicate a more benthic-like body shape while fish with more positive LD1 values have a more limnetic-like body shape.


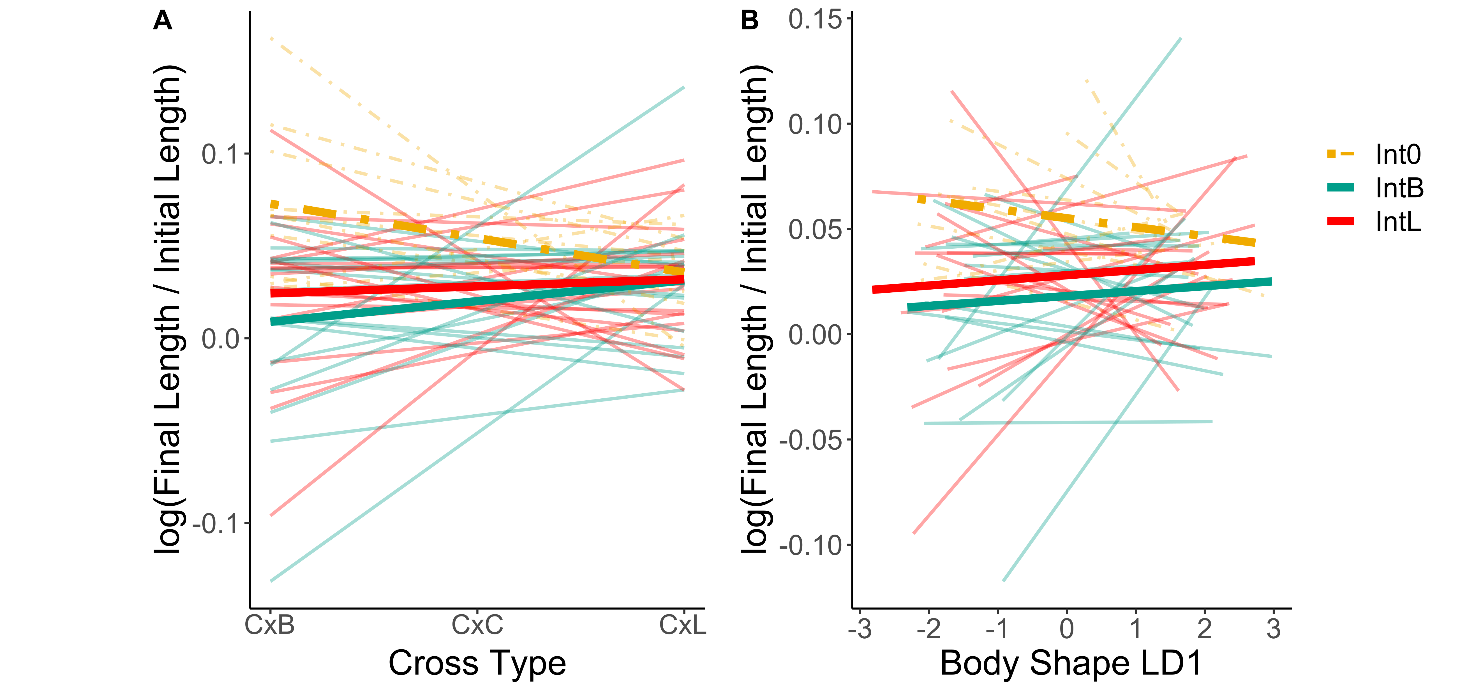


*Fig. S3.* Target population growth in contrasting frequency distributions of phenotypes of both individually- and batch-marked fish. Each line represents one mesocosm. In (A) cross was converted to a numeric value, with CxB = -1, CxC = 0, and CxL = 1. In (B), more negative LD1 values indicate a more benthic-like body shape while fish with more positive LD1 values have a more limnetic-like body shape.


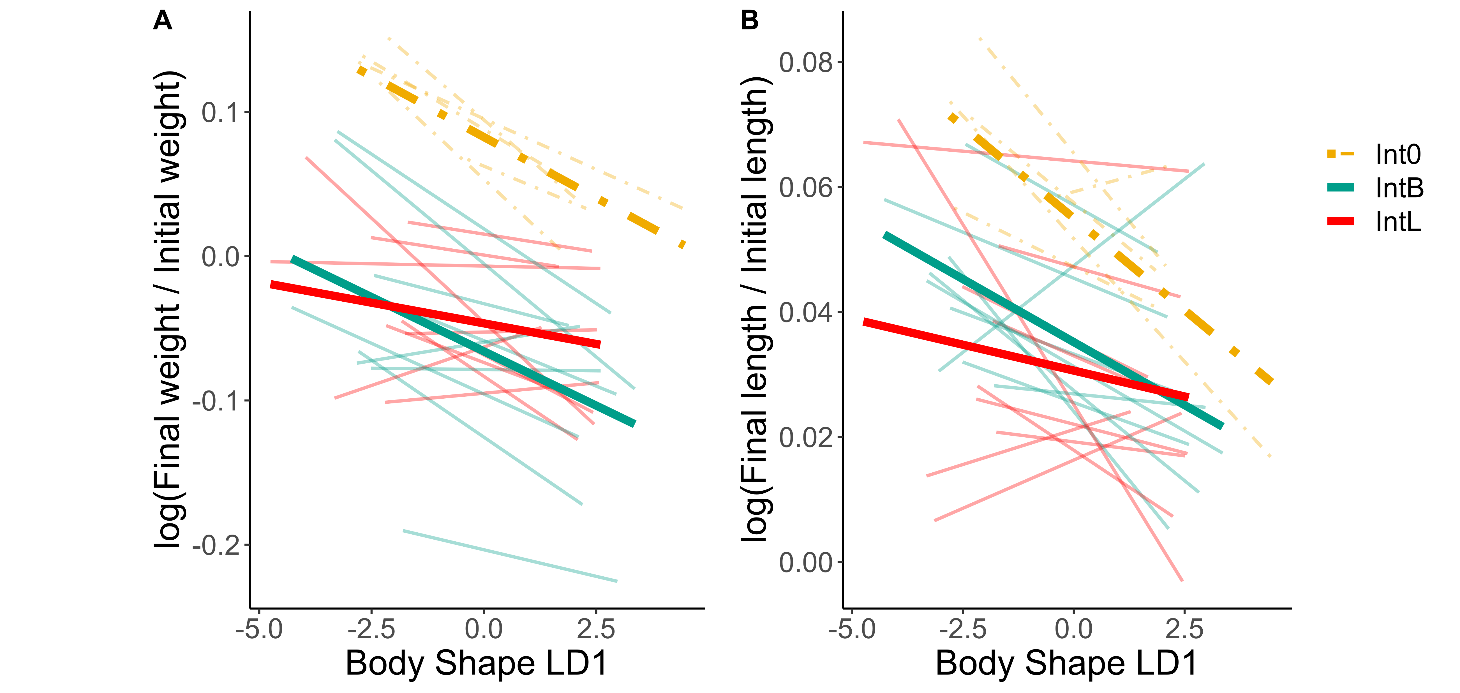


*Fig. S4.* Target population growth in contrasting frequency distributions of phenotypes of individually-marked fish. Each line represents one mesocosm. More negative LD1 values indicate a more benthic-like body shape while fish with more positive LD1 values have a more limnetic-like body shape.

**Supplementary Tables**

Table S1. Tests of the difference between treatment fish presence (Int_L_ and Int_B_) and absence (Int_0_). For dataset = 1, all mesocosms were included. For dataset = 2, we included only mesocosms where all four treatment stage fish were recovered.

| **variable** | **fish** | **dataset** | **mesocosms** | **t-stat** | **df** | **p-value** | **Cohen's D** |
| --- | --- | --- | --- | --- | --- | --- | --- |
| log(final length / initial length) | batch-marked and individually-marked | 1 | 48 | 2.8 | 16.62 | 0.01 | 0.94 |
| log(final length / initial length) | batch-marked and individually-marked | 2 | 34 | 3.15 | 21.25 | <0.01 | 1.13 |
| log(final length / initial length) | individually-marked only | 1 | 25 | 4.99 | 9.05 | <0.01 | 2.19 |
| log(final length / initial length) | individually-marked only | 2 | 18 | 4.77 | 11.22 | <0.01 | 2.28 |
| log(final weight / initial weight) | individually-marked only | 1 | 25 | 8.89 | 23 | <0.01 | 3.01 |
| log(final weight / initial weight) | individually-marked only | 2 | 18 | 8.07 | 15.22 | <0.01 | 3.32 |
| proportion survived | individually-marked only | 1 | 25 | -0.9 | 4.32 | 0.42 | -0.54 |
| proportion survived | individually-marked only | 2 | 18 | -1.08 | 4.53 | 0.34 | -0.65 |

Table S2. Tests of frequency-dependent selection. For tests where slope predictor is "cross type", CxB individuals were coded as -1, CxC individuals were coded as 0, and CxL individuals were coded as 1. For dataset = 1, all mesocosms were included. For dataset = 2, we included only mesocosms where all four treatment stage fish were recovered.

| **slope predictor** | **slope response** | **fish** | **dataset** | **mesocosms** | **t-stat** | **df** | **p-value** | **Cohen's D** |
| --- | --- | --- | --- | --- | --- | --- | --- | --- |
| body shape (LD1) | log(final length / initial length) | batch-marked and individually-marked | 1 | 37 | 1.09 | 32.15 | 0.28 | 0.36 |
| body shape (LD1) | log(final length / initial length) | batch-marked and individually-marked | 2 | 23 | 0.52 | 20.31 | 0.61 | 0.22 |
| cross type | log(final length / initial length) | batch-marked and individually-marked | 1 | 38 | 0.78 | 35.87 | 0.44 | 0.25 |
| cross type | log(final length / initial length) | batch-marked and individually-marked | 2 | 24 | 0.28 | 21.88 | 0.78 | 0.12 |
| body shape (LD1) | log(final length / initial length) | individually-marked only | 1 | 19 | -0.33 | 16.91 | 0.74 | -0.15 |
| body shape (LD1) | log(final length / initial length) | individually-marked only | 2 | 13 | -0.5 | 10.67 | 0.62 | -0.28 |
| body shape (LD1) | log(final weight / initial weight) | individually-marked only | 1 | 19 | -0.84 | 16.95 | 0.41 | -0.38 |
| body shape (LD1) | log(final weight / initial weight) | individually-marked only | 2 | 13 | -0.65 | 10.86 | 0.53 | -0.36 |
| cross type | log(final length / initial length) | individually-marked only | 1 | 20 | -0.04 | 14.32 | 0.97 | -0.02 |
| cross type | log(final length / initial length) | individually-marked only | 2 | 13 | -0.99 | 9.84 | 0.35 | -0.54 |
| cross type | log(final weight / initial weight) | individually-marked only | 1 | 20 | 1.25 | 16.93 | 0.23 | 0.56 |
| cross type | log(final weight / initial weight) | individually-marked only | 2 | 13 | 0.8 | 10.97 | 0.44 | 0.44 |
| mean LD1 for each cross type | proportion survived | individually-marked only | 1 | 19 | 1.96 | 16.89 | 0.07 | 0.89 |
| mean LD1 for each cross type | proportion survived | individually-marked only | 2 | 13 | 1.06 | 9.94 | 0.31 | 0.58 |
| cross type | proportion survived | individually-marked only | 1 | 20 | 2.34 | 14.69 | 0.03 | 1.05 |
| cross type | proportion survived | individually-marked only | 2 | 13 | 1.45 | 8.92 | 0.18 | 0.79 |

Table S3. Tests of differences in slope between fish presence and absences. For tests where slope predictor is "cross type", CxB individuals were coded as -1, CxC individuals were coded as 0, and CxL individuals were coded as 1. For dataset = 1, all mesocosms were included. For dataset = 2, we included only mesocosms where all four treatment stage fish were recovered.

| **slope predictor** | **slope response** | **fish** | **dataset** | **mesocosms** | **t-stat** | **df** | **p-value** | **Cohen's D** |
| --- | --- | --- | --- | --- | --- | --- | --- | --- |
| body shape (LD1) | log(final length / initial length) | batch-marked and individually-marked | 1 | 47 | -2.39 | 13.50 | 0.03 | -0.87 |
| body shape (LD1) | log(final length / initial length) | batch-marked and individually-marked | 2 | 33 | -1.85 | 16.72 | 0.08 | -0.70 |
| cross type | log(final length / initial length) | batch-marked and individually-marked | 1 | 48 | -2.60 | 15.34 | 0.02 | -0.90 |
| cross type | log(final length / initial length) | batch-marked and individually-marked | 2 | 34 | -2.03 | 20.13 | 0.056 | -0.74 |
| body shape (LD1) | log(final length / initial length) | individually-marked only | 1 | 24 | -1.06 | 5.87 | 0.33 | -0.55 |
| body shape (LD1) | log(final length / initial length) | individually-marked only | 2 | 18 | -0.95 | 7.93 | 0.37 | -0.49 |
| body shape (LD1) | log(final weight / initial weight) | individually-marked only | 1 | 24 | -2.63 | 9.66 | 0.03 | -1.16 |
| body shape (LD1) | log(final weight / initial weight) | individually-marked only | 2 | 18 | -2.11 | 12.82 | 0.05 | -0.98 |
| cross type | log(final length / initial length) | individually-marked only | 1 | 25 | -0.54 | 5.95 | 0.61 | -0.28 |
| cross type | log(final length / initial length) | individually-marked only | 2 | 18 | -0.61 | 7.22 | 0.56 | -0.32 |
| cross type | log(final weight / initial weight) | individually-marked only | 1 | 25 | 1.06 | 12.60 | 0.31 | 0.43 |
| cross type | log(final weight / initial weight) | individually-marked only | 2 | 18 | 1.12 | 15.20 | 0.28 | 0.49 |
| mean LD1 for each cross type | proportion survived | individually-marked only | 1 | 24 | 2.25 | 5.85 | 0.07 | 1.17 |
| mean LD1 for each cross type | proportion survived | individually-marked only | 2 | 18 | 2.27 | 6.06 | 0.06 | 1.25 |
| cross type | proportion survived | individually-marked only | 1 | 25 | 2.30 | 7.81 | 0.05 | 1.06 |
| cross type | proportion survived | individually-marked only | 2 | 18 | 2.18 | 9.40 | 0.06 | 1.08 |

Table S4. Population history and number of treatment fish recovered between treatment and target population phases.

| **Mesocosm** | **Treatment** | **Lake** | **Recovered** |
| --- | --- | --- | --- |
| T1-3 | IntB | Bullocks | 4 |
| T3-6 | IntB | Bullocks | 4 |
| T3-8 | IntB | Bullocks | 4 |
| T4-7 | IntB | Bullocks | 4 |
| T5-1 | IntB | Bullocks | 4 |
| T6-3 | IntB | Bullocks | 4 |
| T1-7 | IntB | Bullocks | 3 |
| T2-8 | IntB | Bullocks | 3 |
| T4-8 | IntB | Bullocks | 2 |
| T4-5 | IntB | Bullocks | 1 |
| T1-2 | IntB | Hoggan | 4 |
| T4-3 | IntB | Hoggan | 4 |
| T4-6 | IntB | Hoggan | 4 |
| T4-9 | IntB | Hoggan | 4 |
| T5-2 | IntB | Hoggan | 4 |
| T5-4 | IntB | Hoggan | 4 |
| T1-5 | IntB | Hoggan | 3 |
| T2-4 | IntB | Hoggan | 0 |
| T2-5 | IntB | Hoggan | 0 |
| T6-1 | IntB | Hoggan | 0 |
| T2-7 | IntL | Ambrose | 4 |
| T4-2 | IntL | Ambrose | 4 |
| T5-9 | IntL | Ambrose | 4 |
| T6-2 | IntL | Ambrose | 2 |
| T2-2 | IntL | Garden Bay | 4 |
| T1-4 | IntL | Little Quarry | 4 |
| T5-7 | IntL | Little Quarry | 4 |
| T2-10 | IntL | Little Quarry | 3 |
| T4-1 | IntL | Little Quarry | 3 |
| T2-1 | IntL | North | 4 |
| T3-4 | IntL | North | 4 |
| T5-8 | IntL | North | 4 |
| T2-3 | IntL | North | 3 |
| T2-9 | IntL | North | 3 |
| T3-9 | IntL | Paq | 4 |
| T4-4 | IntL | Paq | 4 |
| T1-9 | IntL | Paq | 3 |
| T1-6 | IntL | Paq | 2 |
| T3-5 | IntL | Priest | 4 |
| T3-7 | IntL | Priest | 4 |
